# Epicatechin Used in the Treatment of Intestinal Inflammatory Disease: An Analysis by Experimental Models

**DOI:** 10.1155/2012/508902

**Published:** 2012-12-30

**Authors:** Paulo César de Paula Vasconcelos, Leonardo Noboru Seito, Luiz Cláudio Di Stasi, Clélia Akiko Hiruma-Lima, Cláudia Helena Pellizzon

**Affiliations:** ^1^Morphology Department, Biosciences Institute, UNESP-University Estadual Paulista, P.O. Box 510, 18618-970 Botucatu, SP, Brazil; ^2^Pharmacology Department, Biosciences Institute, UNESP-University Estadual Paulista, P.O. Box 510, 18618-970 Botucatu, SP, Brazil; ^3^Physiology Department, Biosciences Institute, UNESP-University Estadual Paulista, P.O. Box 510, 18618-970 Botucatu, SP, Brazil

## Abstract

*Background*. This study was pathway of (−)-epicatechin (EC) in the prevention and treatment of intestine inflammation in acute and chronic rat models. *Methods*. Intestine inflammation was induced in rats using TNBS. The morphological, inflammatory, immunohistochemical, and immunoblotting characteristics of colon samples were examined. The effects of EC were evaluated in an acute model at doses of 5, 10, 25, and 50 mg/kg by gavage for 5 days. The chronic colitis model was induced 1st day, and treated for 21 days. For the colitis relapse model, the induction was repeated on 14th. *Results*. EC10 and EC50 effectively reduced the lesion size, as assessed macroscopically; and confirmed by microscopy for EC10. The glutathione levels were higher in EC10 group but decreased COX-2 expression and increased cell proliferation (PC) were observed, indicating an anti-inflammatory activity and a proliferation-stimulating effect. In the chronic colitis model, EC10 showed lower macroscopic and microscopic lesion scores and increase in glutathione levels. As in the acute model, a decrease in COX-2 expression and an increase in PC in EC10, the chronic model this increase maybe by the pathway EGF expression. *Conclusion*. These results confirm the activity of EC as an antioxidant that reduces of the lesion and that has the potential to stimulate tissue healing, indicating useful for preventing and treating intestine inflammation.

## 1. Introduction

Inflammation of the colon can be caused by a variety of illnesses and infections. Inflammatory bowel disease (IBD) is a group of inflammatory conditions of the colon and small intestine, which includes mainly ulcerative colitis and Crohn's disease. The etiology of IBD remains poorly understood. It is postulated to be related to poor blood supply, autoimmune reactions and to be predisposed by infections. Ulcerative colitis, the most similar disease to our experimental model, is indicated in some studies to be caused by the deregulation of the mucosal immune system and pathological T cell responses in genetically susceptible individuals [1], classifying it as an autoimmune disease. Another hypothesis suggests that ulcerative colitis begins as a disorder of the mucosal barrier and that this disorder may be the initial factor that allows subsequent attacks by colonic commensal bacteria to cause inflammation of the mucosa [2].

The currently available therapies against ulcerative colitis include glucocorticosteroids, sulfasalazine, 5-aminosalicylic acid, immunosuppressive agents, and anti-TNF-*α* monoclonal antibodies; in some cases, colostomy surgery is the only alternative [3]. These treatments are not free from side effects, which may become significant due to the need for chronic treatment, which is common. Therefore, the search for alternative treatments is crucial, and the ability to provide effective cytoprotection for the region of inflammation is of particular importance. With this in mind, we tested (−)-epicatechin, a substance found in many flavonoid-rich plants, such as *Mouriri pusa* Gardn. (Melastomataceae), which was previously studied in our laboratory [4, 5]. Among the substances found in this plant extract, epicatechin was chosen as the subject of this study because it is considered an important cytoprotectant due to its strong antioxidant activity and its inhibition of apoptosis by interfering in the caspase signaling chain [6]. Galvez and collaborators [7] found that epicatechin potently inhibited lipid peroxidation in both enzymatic and nonenzymatic models of colitis involving arachidonic acid *in vitro*; this inhibition was independent of its effect on the glutathione enzymatic system. Thus, epicatechin may play an important role in removing free radicals from the inflamed mucosa of the colon, thus aiding the recovery process. Furthermore, epicatechin may inhibit the peroxidation mediated by arachidonic acid, indicating its potential as an anti-inflammatory agent in IBD, by affecting the activity of enzymes such as lipoxygenase and cyclooxygenase. 

A role for epicatechin in the prevention of cancer was also reported; epicatechin promotes the maintenance of gap junctions between epithelial cells [8], helping to prevent the progression of gastrointestinal lesions into malignant lesions.

We aimed to identify pharmacologically active substances through the study of medicinal species to provide new therapeutic options for gastrointestinal diseases, thereby contributing to the sustainable development of the flora of the Cerrado of Sao Paulo state, Brazil. The aim of this study is to assess the role of (−)-epicatechin in the prevention and treatment of intestine inflammation in rats through macroscopic, microscopic and biochemical analyses of the intestinal lesions in acute and chronic models of colitis. The anti-inflammatory and healing effects of (−)-epicatechin will be assessed.

## 2. Materials and Methods

### 2.1. Animals

Male Wistar rats (200–250 g) from the Central Animal House of UNICAMP were used. The rats were fed a certified diet, had free access to tap water and were kept under standard lighting (12 h dark–12 h light), humidity (60 ± 1%), and temperature (21 ± 2°C) conditions. All experimental protocols followed the recommendations of the Animal Experimentation Ethic Committee of the Bioscience Institute of UNESP—Botucatu, Sao Paulo state, Brazil, by Protocol 02/04.

### 2.2. Induction Model and Treatment of Intestine Inflammation

The chosen model of intestinal inflammation consisted of the intracolonic administration of trinitrobenzene sulfonic acid (TNBS) dissolved in 0.25 ml of 50% ethanol/water in rats [9]. The doses of (−)-epicatechin used (between 5 and 50 mg/kg) were selected based on the doses of other phenolic compounds, such as other flavonoids and paepalantine, that have demonstrated anti-inflammatory activities in this experimental model [10, 11].

A TNBS control group (with induction of colonic inflammation, but without pharmacological treatment) and a noncolitic control group (without induction of inflammation) were included in each experiment, along with a positive treatment group of animals (*n* = 7) that were treated with sulfasalazine (100 mg/kg). All drugs were dissolved in 10% alcoholic saline solution. The general protocol of the experiments involved two treatments, acute and chronic with relapse, which are described below.

#### 2.2.1. Acute Colitis

Four groups of animals received different doses (5, 10, 25, and 50 mg/kg) of (−)-epicatechin orally once per day for two days before the induction of colitis, as well as 2 hours before and 24 hours after the induction of colitis. The (−)-epicatechin was dissolved in 10% alcoholic saline solution and administered by gavage. The animals from the TNBS control and from the noncolitic group received only the vehicle (10 ml/kg). The positive control group received sulfasalazine (100 mg/kg) dissolved in the same vehicle. The body weight and occurrence of diarrhea was monitored daily for each group.

#### 2.2.2. Relapsed Colitis

In this three-week experimental protocol, colitis was first induced with 10 mg of TNBS in 50% ethanol, as previously described, and the animals received a second dose of 10 mg of TNBS after 14 days [12]. The animals were divided into 5 groups: two groups received daily doses of 10 mg/kg (EC10) or 50 mg/kg epicatechin (EC50), respectively; these doses were selected based on their efficacy in the acute colitis experiment. The other groups were as follows: noncolitic (no colitis nor relapse was induced; animals received 10 mL/kg vehicle), TNBS control (colitis and relapse were induced; animals received 10 mL/kg vehicle), sulfasalazine (colitis and relapse were induced; animals received 100 mg/kg of sulfasalazine), and TNBS control without relapse (colitis, but not relapse, was induced; animals received 10 mL/kg vehicle). One-third of the animals from each group were sacrificed one week after the induction of colitis with TNBS, one-third were sacrificed after two weeks, and the remaining one-third (those submitted to relapse) were sacrificed after three weeks. The TNBS control without relapse group was entirely sacrificed after three weeks.

### 2.3. Assessment of the Intestinal Inflammatory Process

During the course of the experiments, animals were assessed using various markers of overall health, such as food consumption, body weight, and the appearance of diarrhea. At the end of each treatment, the animal was sacrificed, and its colon was removed and assessed for damage using macroscopic, biochemical, microscopic, immunohistochemical, and immunoblotting methods.

### 2.4. Macroscopic Assessment

The weight of the colons, the adherence of the intestine to adjacent organs and the severity and extent of intestinal damage were assessed as described by Bell and colleagues [13].

### 2.5. Biochemical Assessment

Tissue samples from the colons were assessed for total glutathione [14], myeloperoxidase (MPO) activity [12], alkaline phosphatase [15], and total protein content using the bicinchoninic acid (BCA) method [16]. 

### 2.6. Microscopic Assessment

Immediately after the macroscopic assessment of the colon tissue, samples of the tissue (0.5 mm) adjacent to the focus of the lesion were taken and processed for histological analysis; 6 *μ*m slices were taken, and the slides were stained with hematoxylin and eosin. Microscopic assessment was recorded on a scale from 0 (absence of lesion) to 27, as described by Stucchi and colleagues [17] and modified by Camuesco and colleagues [18].

### 2.7. Immunohistochemistry

The samples in which the greatest effect was observed, based on the previously measured parameters, were assessed histologically. To prepare the tissue for histological assessment, samples were deparaffinized, rehydrated, and submitted to antigen recovery using 0.1 M citrate buffer in a microwave oven at high temperature for 10 minutes. After antigen recovery, the slides were placed in blocking solution for 30 minutes. The slides were then placed in primary antibody diluted in PBS solution. The primary antibodies used were either COX-2 (Santa Cruz), to assess the inflammation level [19–21], or PCNA (Proliferating Cell Nuclear Antigen) (Santa Cruz), a cell division marker, to assess the level of tissue regeneration. The samples were then washed in PBS, incubated with biotinylated secondary antibody, washed again, and then incubated with the ABC reagent (avidin-biotin peroxidase complex) by Vectastain and incubated with DAB (diaminobenzidine). The resulting slides were analyzed using a LEICA microscope; 0.32 mm^2^ fields were captured using Leica Q-Win software. The quantification was performed using the AVSoft BioView 4 software.

### 2.8. Electrophoresis and Immunoblotting Analysis

Protein samples from the intestines were quantified using the Bradford method [22] and approximately 50 *μ*g of protein per lane was resolved via 7.5% sodium dodecyl sulfate-polyacrylamide gel electrophoresis (SDS-PAGE). After migration, the proteins were processed for immunoblotting. For samples from the acute colitis model, anti-HSP-70 (Heat Shock Protein 70) primary antibody [23, 24] was used. For samples from the model of chronic colitis with relapse, EGF (Epidermal Growth Factor) [25] and iNOS (inducible Nitric Oxide Synthase) [26, 27] antibodies were used. 

### 2.9. Statistical Analysis

The results were expressed as the mean ± standard error of the mean. Differences between the means were tested by analysis of variance (ANOVA) followed by tests of significance. Nonparametric data (scores) were expressed as median and interquartile range and were analyzed by the Mann-Whitney *U* test. Frequency data were analyzed by the *χ* ^2^ test. Values of *P* < 0.05 were considered to be statistically significant.

## 3. Results 

### 3.1. Induction and Treatment of Acute Colitis

After the induction of acute colitis, a score was given to each sample based on the macroscopic analysis of the lesions. These scores revealed the effectiveness of 10 mg/kg (EC10) and 50 mg/kg (EC50) doses of (−)-epicatechin in diminishing the severity of lesions (Table 1) compared with the non-colitis group. These results were confirmed by a microscopic analysis of the lesions in the group treated with the EC10 dose (Table 1).

The induction of colitis caused a decrease in body weight gain, an increase in the weight of the colons and a decrease in the length of the colons. These changes were similar in all of the groups in which colitis had been induced, regardless of the treatment (data not shown).

**Figure 1:**
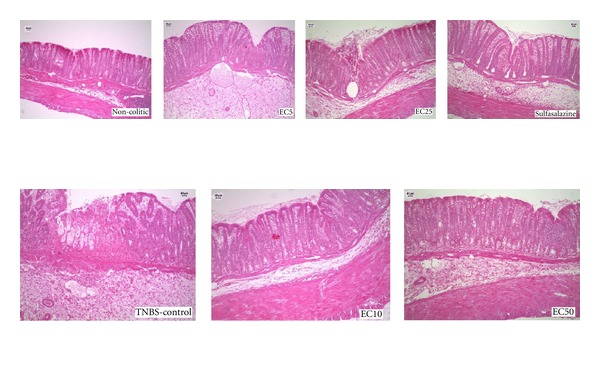
Histological analysis of colons from rats subjected to acute experimental colitis. The samples were stained with Hemantoxylin and Eosin.

Histological analyses of the TNBS controls showed ulceration and disorganization of the mucosa and increased edema in the lamina propria and submucosa (Figure 1). In the groups treated with EC10 and EC50, the morphological organization of the tissue was closer to that in the noncolitic group, exhibiting less ulceration and disorganization than TNBS controls. The groups treated with EC5 and EC25 showed ulceration and increased edema of the lamina propria and submucosa.

### 3.2. Biochemical Analysis of the Acute Model of Colitis

The biochemical analyses provided information regarding the probable mechanisms of action of EC in colon tissue from rats treated with EC10 (which had reduced macroscopic and microscopic scores). This group presented significantly higher levels of glutathione than the TNBS controls (Table 2). No difference was found between the treated colitis group and the TNBS controls with regard to the other parameters analyzed (MPO, alkaline phosphatase, and total protein) (Table 2). 

### 3.3. Immunolabeling Analysis of the Acute Model of Colitis

**Figure 2:**
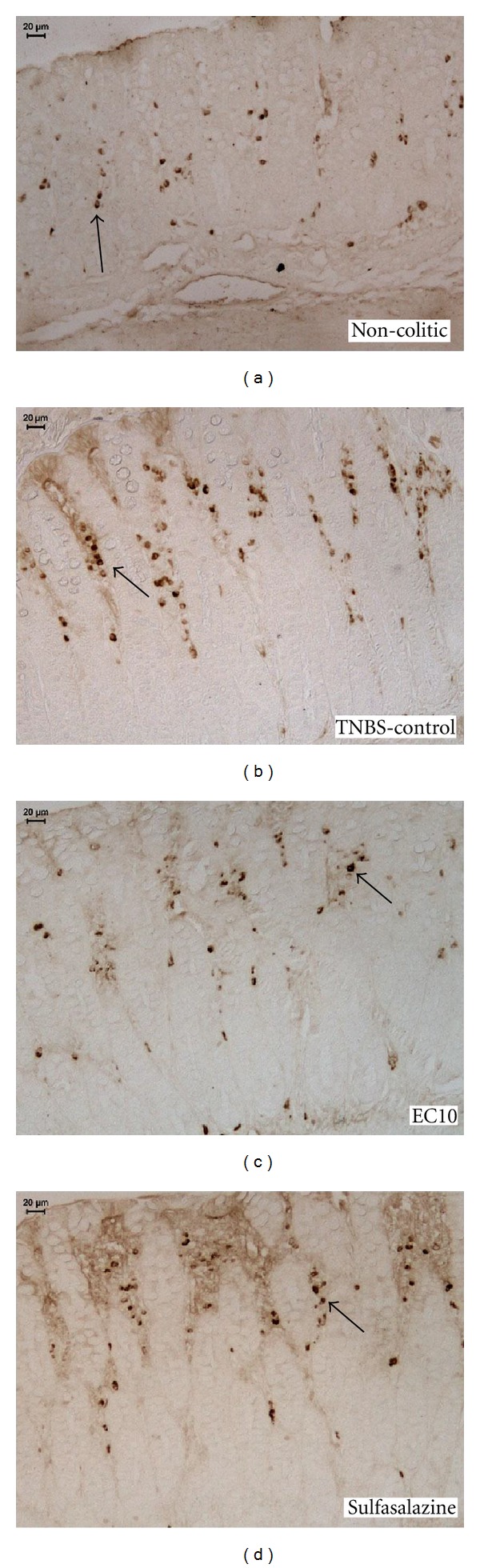
Immunohistochemical analysis of expression of COX-2 in the colons of rats from different treatments subjected to acute experimental colitis. Arrows indicate expression.

**Figure 3:**
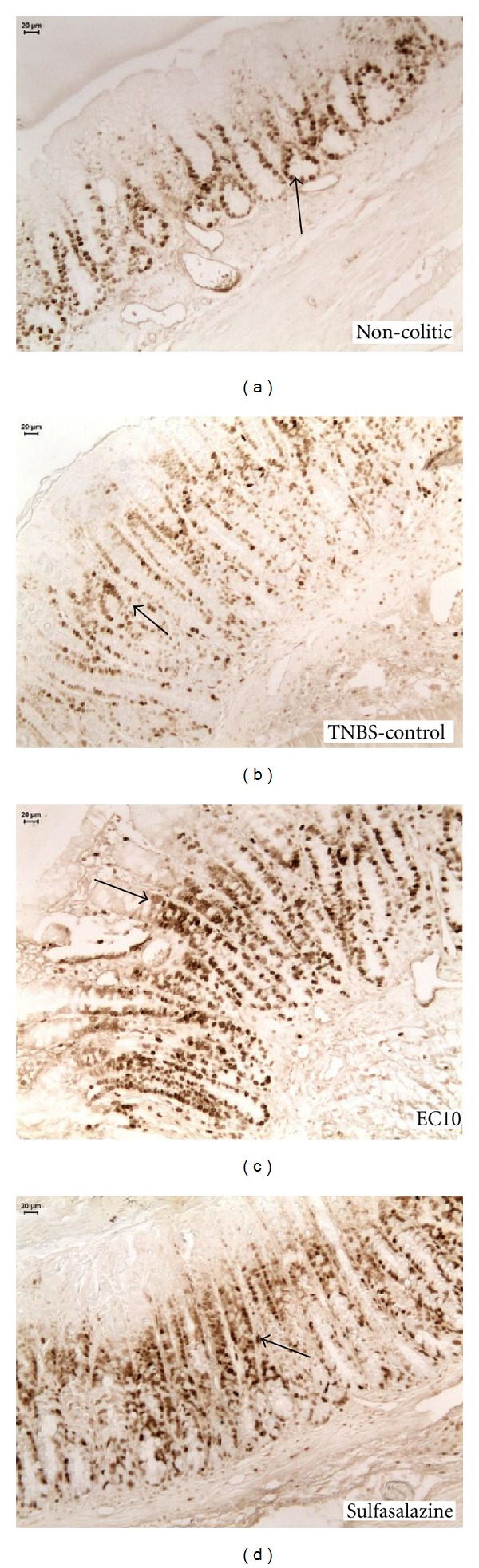
Immunohistochemical analysis of expression of PCNA-positive cells in the colons of rats from different treatments subjected to acute experimental colitis. Arrows indicate expression.

Immunohistochemistry against COX-2 (Figure 2) revealed that EC10 (the only dose assessed because it provided the greatest effect) caused a significant decrease in COX-2 expression compared with the TNBS controls (Table 3). Antibody labeling of PCNA (Figure 3) showed an increased expression of this peptide in the EC10 group compared with the TNBS control group (Table 3).

**Figure 4:**
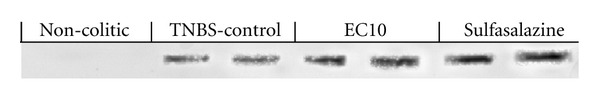
Immunoblotting and measurement of HSP-70 in the colons of rats from different treatments subjected to acute experimental colitis.

No difference in the expression of HSP-70 between the samples from the EC10 and TNBS control groups was observed by immunoblotting (Figure 4).

### 3.4. Induction and Treatment of Chronic Colitis with Relapse

We used two treatment groups to assess chronic colitis based on the observation that the 10 and 50 mg/kg doses of EC had the best therapeutic effects in the acute assays. At the end of each week of the three-week assay, the macroscopic scores of the lesions were recorded. As in the acute assay, the group treated with EC10 had significantly lower scores than did the TNBS control group (Table 4), including those occurring after relapse. In the third week (after relapse), the score of the group treated with EC10 was not significantly different from that of the TNBS control group without relapse in contrast to those of the other colitic groups.

The mean body weights were not significantly different between the groups treated with epicatechin and the noncolitic group (data not shown). With regard to the weight and the length of the colon in the chronic experiment, the noncolitic group had longer colons, as in the acute assay, but only after the second and third weeks. The EC group did not differ from the TNBS control group at any point. 

**Figure 5:**
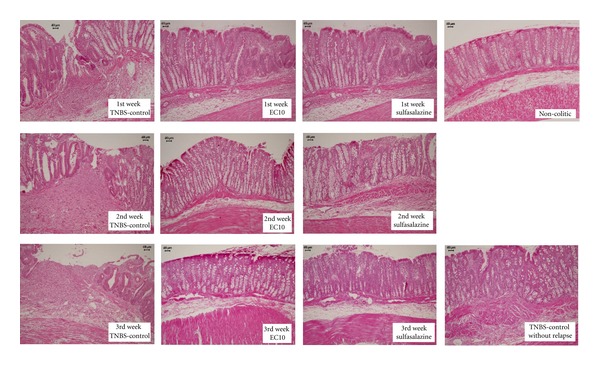
Histological analysis of colons from rats subjected to chronic experimental colitis with relapse. The samples were stained with Hemantoxylin and Eosin.

The histological analysis of tissues from the EC10 group showed that the integrity of the mucosa was maintained, and little edema of the mucosa and submucosa was present, as observed in the noncolitic group. The analysis of TNBS controls showed ulcerated areas and extensive edema (Figure 5).

### 3.5. Biochemical Analysis of the Model of Chronic Colitis with Relapse Assay

As in the acute assay, the biochemical quantifications revealed a significant increase in glutathione levels in the group treated with EC10 compared with the TNBS controls in the first and third weeks. This confirmed that EC acts as an antioxidant by maintaining levels of glutathione in the tissue (Table 5). In the second week, no increase was detected, due to the longer elapsed time since the induction (two weeks) than in either the first (one week) or third (one week after relapse) weeks. No significant difference in myeloperoxidase or alkaline phosphatase activity was observed between the EC groups and the TNBS control.

### 3.6. Immunolabeling Analysis for the Chronic Model

**Figure 6:**
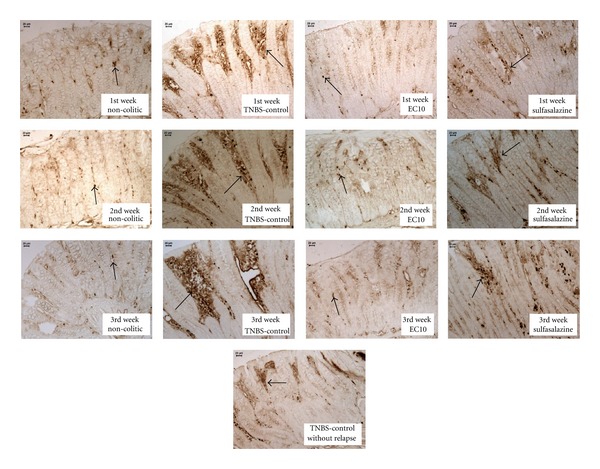
Immunohistochemical analysis of expression of COX-2 in the colons of rats from different treatments subjected to chronic experimental colitis with relapse. Arrows indicate expression.

**Figure 7:**
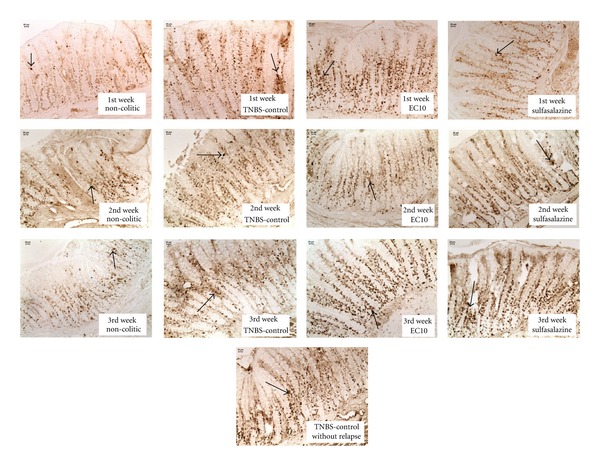
Immunohistochemical analysis of expression of PCNA-positive cells in the colons of rats from different treatments subjected to chronic experimental colitis with relapse. Arrows indicate expression.

As in the acute assay, there was a decrease in the expression of COX-2 (Figure 6) in the colon tissue of the EC10-treated group, as evaluated by immunohistochemistry (Table 6). For all three weeks analyzed, the expression of COX-2 was significantly lower in the EC10 group than in the TNBS controls, confirming the anti-inflammatory activity observed in the acute assay. Significantly higher levels of PCNA positive cells (Figure 7) were observed in EC10 than in the TNBS control in the 1st and 3rd weeks (Table 6).

**Figure 8:**
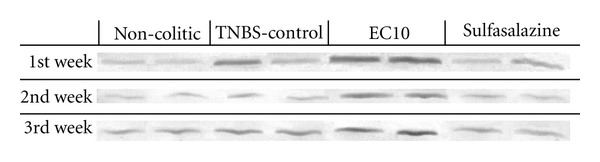
Immunoblotting and measurement of EGF levels after induction of chronic colitis with relapse.

**Figure 9:**
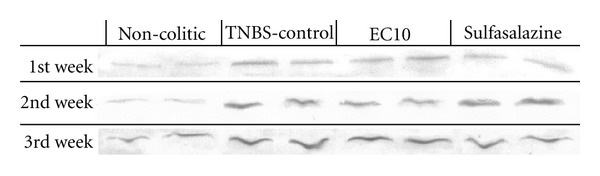
Immunoblotting and measurement of iNOS levels after induction of chronic colitis with relapse.

To investigate the mechanism underlying the increase in cell proliferation, the presence of EGF in the mucosa of the colons was determined by immunoblotting. It was found that the colons from the EC10 group had higher levels of EGF than those in the TNBS controls (Figure 8). This difference in EGF levels could explain the increase in cell proliferation caused by EC treatment. The expression of iNOS was also assessed by immunoblotting; none of the treatments used in this study reduced the expression of iNOS (Figure 9).

## 4. Discussion 

The induction of intestine inflammation by TNBS provides an effective animal model of colitis. Animals subjected to TNBS-induced colitis suffered from diarrhea, weight loss (in the special TNBS control group), and the disorganization of the mucosa and submucosa of the colon; the changes measured in these parameters are similar to those obtained by Luchini and collaborators [28], who tested coumarin and 4-hydroxycoumarin. 

One of the most interesting findings in this study regards glutathione (GSH). GSH is a tripeptide that is produced by mucosal cells and functions as an antioxidant during oxidative metabolism; its roles include the removal of hydroperoxides and the maintenance of the physiological state of protein sulfhydryl groups [29, 30]. As EC10 led to higher GSH levels in both acute and chronic with relapse models, it is possible to infer that epicatechin stimulates GSH expression in the tissue, thereby acting as an antioxidant. It is also possible that epicatechin has a direct antioxidant action that prevents the depletion of GSH by the inflammatory process. This observation is corroborated by the preservation of the epithelial architecture in the EC-treated group.

Despite its ability to decrease the infiltration by neutrophils (as observed by microscopy), epicatechin was unable to reduce the levels of MPO in the colon tissue compared with the TNBS controls. This inability may be related to the nature of the induction of colitis used, which causes such an extensive damage that MPO high levels are strongly sustained, so that it is uncommon for any treatment to exhibit this pharmacological activity [31]. That inability was compensated by the higher levels of GSH found in the colons of animals treated with epicatechin, that, together with the ability of epicatechin to inhibit lipid peroxidation *in vitro,* independently of its interference in the GSH activity [7], supports the hypothesis that epicatechin acts to normalize oxidative stress by antioxidant mechanisms. This beneficial activity on intestine inflammation is frequently observed following treatment with plant extracts that are rich in polyphenol compounds (such as *Turnera ulmifolia* [32]) due to their antioxidant activity. The imbalance between the formation of oxygen-reactive species and anti-oxidative micronutrients is important in the pathogenesis and perpetuation of tissue damage in inflammatory diseases in general [33]. Therefore, therapies that use an antioxidant approach may be promising in the treatment and prevention of ulcerative colitis.

Anti-inflammatory activity is also of interest for the treatment of IBD. Flavonoids are recognized as anti-inflammatory compounds, and their therapeutic effects in protecting the mucosa are exerted through a complex mechanism involving the inhibition of eicosanoid synthesis and/or the clearance of free radicals and antioxidant activity [34]. Several studies have shown that the flavonoids from *Mouriri pusa* caused a decrease in the expression of COX-2 in the gastric mucosa [5, 34]. A similar effect was observed in this study, highlighting one probable mechanism of action of EC.

Lesion healing was assessed by the quantifications of PCNA and EGF. PCNA positive cells are in the S phase of the cell cycle, so this antigen may be used as a marker for proliferating cells [35]. In the colon, the number of crypt cells is strictly regulated, and the balance between cell proliferation and cell death is required to maintain homeostasis [36]. In the PCNA immunolabeling assay, we observed an increase in PCNA labeling in the EC groups in the acute experimental model. In the chronic model, although the PCNA level was increased in the EC10 group at the first and third weeks of treatment, in the second week, there was no significant alteration, most likely because the lesions had healed significantly. This cell proliferation is highly beneficial for the regeneration of the intestinal mucosa because the new cells replace damaged epithelial cells.

The increase in EGF expression observed in our work may explain the increased cell proliferation observed, and may be one of the mechanisms of action of EC10 against colitis. EGF is known to stimulate epithelial cell proliferation in a variety of systems [37]; indeed many growth factors modulate cell proliferation, cell differentiation, angiogenesis, inflammation, and gastrointestinal defense mechanisms. This is exemplified by intestinal wound repair *in vitro* and *vivo* [38]. The administration of EGF by enema in conjunction with oral mesalazine treatment significantly reduced colitic inflammation compared to mesalazine treatment and a placebo enema [39]. The ulceration of the epithelium induces the differentiation of a gastrointestinal stem cell strain that grows locally as a tubule adjacent to the ulcer, producing and secreting EGF [40]. In this case, EGF stimulates cell proliferation and the regeneration of the intestinal mucosal epithelium [25].

The model of chronic colitis has provided important evidence for the therapeutic effect of epicatechin because the development of the relapse lesion for the EC10 group was inefficient, indicating that epicatechin has the potential to prevent the exacerbation of the colitis lesion due to a relapse of the harmful stimulus. Consistent with this hypothesis, the microscopic analysis of the lesions revealed that the EC10 group had a significantly lower score than the TNBS control group in the three weeks assessed and was not significantly different from the TNBS controls without relapse.

In this study, the beneficial effect of the 10 mg/kg dose of epicatechin upon the intestinal lesions disappeared when the dose was increased, a phenomenon previously demonstrated with other phenolic substances used in the same disease model. This suggests that, at higher doses, the pro-oxidant action of such substances outweighs their beneficial effects on intestine inflammation [41].

In an attempt to elucidate other mechanisms, proteins such as HSP-70 in the acute model and iNOS in the chronic model were also quantified. HSP-70 (Heat Shock Protein 70) is an essential protein for maintaining intestinal homeostasis in colitis [42]. This protein is more abundant, conserved and consistently produced in cells in response to various forms of stress [43] such as heat, toxic agents, infection, and proliferation [44]. In addition, there is evidence that HSP-70 plays a critical role in protecting the colonic mucosa from colitis-induced stress [45]. Figure 2, which shows the expression of HSP-70, indicates that this protein is neither increased as a mechanism of EC10 action nor decreased as a diminution of cell sensitivity to stress; this was also observed with other flavonoids such as quercetin [46].

The expression of iNOS (inducible nitric oxide synthase) was also assessed by immunoblotting because it is an enzyme with an important role in the pathogenesis of ulcerative colitis [27], and its expression is increased in the colitic colon [47]. The anti-inflammatory effect of the flavonoid quercetin on ulcerative colitis may be related to its ability to inhibit the expression of iNOS. However, although the TNBS controls exhibited an increase in the expression of iNOS compared with the noncolitic group [18], none of the treatments reduced this expression of iNOS. 

## 5. Conclusion

These results provide evidence that 10 mg/kg epicatechin effectively reduced lesion severity in both acute and chronic models of colitis. Although epicatechin did not decrease neutrophil infiltration in the mucosa, as shown by the lack of change in the concentration of the enzyme MPO, epicatechin protected the mucosa against the damage from inflammatory infiltration by decreasing the oxidative stress, as observed by the maintenance of glutathione levels. The anti-inflammatory action of epicatechin was also observed in the reduction in the levels of COX-2 in the tissue. Furthermore, epicatechin was able to stimulate cell proliferation and reparation of the epithelium by stimulating the expression of EGF. Growth factors such as EGF have been increasingly used in the treatment of intestinal inflammatory diseases and reveal a potential target for the development of new drugs.

## Figures and Tables

**Table 1:** Macroscopic and microscopic scores of the different groups submitted to the acute model.

	Non-colitic	TNBS control	EC 5	EC 10	EC 25	EC 50	Sulfa.
Macroscopic score	0*	9 (1.25)	8 (1)	7 (1)*	7.5 (1)	7 (0.5)*	8 (1.5)
Microscopic score	0*	19 (1.5)	18 (1.0)	15 (1.5)*	18 (3.5)	16 (2.0)	16 (1.0)

Results expressed as median (interquartile range). Kruskal-Wallis-Mann-Whitney, *P < 0.05 in relation to the check. *n* = 5–8. EC 5 to 50: Epicatechin, doses 5 to 50 mg/kg; Sulfa.: Sulfasalazine.

**Table 2:** Biochemical quantifications of glutathione, myeloperoxidase (MPO), and alkaline phosphatase in the colon of the animals submitted to the acute colitis model.

	Glutathione (nmol/g)	MPO (U/g)	Alkaline phosphatase (mU/mg of protein)
Non-colitic	1,555.75 ± 107.83*	16.98 ± 4.63*	8.43 ± 0.79*
TNBS control	954.73 ± 69.74	1,780.19 ± 263.77	17.21 ± 1.16
EC 5	1,035.49 ± 72.95	1,975.90 ± 294.86	20.78 ± 1.98
EC 10	1,176.55 ± 46.34*	1,876.74 ± 243.27	17.83 ± 1.30
EC 25	1,130.95 ± 42.46	1,973.38 ± 178.27	20.04 ± 2.71
EC 50	1,127.25 ± 35.36	1,806.57 ± 219.35	20.47 ± 2.02
Sulfasalazine	1,104.60 ± 22.33	1,814.55 ± 214.46	20.49 ± 2.01

Results are mean ± E.P.M. ANOVA-Dunnet comparing to the TNBS control group, *P < 0.05. *n* = 5–8. EC 5 to 50: Epicatechin, doses 5 to 50 mg/kg.

**Table 3:** Immunohistochemical quantifications of COX-2 and PCNA-positive cells and immunoblotting of HSP-70 in the colon of the animals submitted to the acute colitis model.

	COX-2 (*μ*m^2^/mm^2^)	PCNA (cells/mm^2^)	HSP-70 (RU)
Non-colitic	4,058.65 ± 3356.31*	249.21 ± 18.49*	4.61
TNBS Control	24,207.99 ± 2614.22	399.34 ± 19.43	335.70
EC 10	9,257.06 ± 3110.30*	572.83 ± 15.69*	456.28
Sulfasalazine	14,635.04 ± 2039.01*	372.52 ± 24.85	541.07

Results are mean ± E.P.M. ANOVA-Dunnet. *P < 0.05 comparing to the TNBS control. *n* = 5–8. EC 10: Epicatechin, dose 10 mg/kg. RU: random units.

**Table 4:** Macroscopic and microscopic scores of the colitic lesions of different groups submitted to the chronic colitis model with relapse.

	1st week score		2nd week score		3rd week score	
	Macro	Micro	Macro	Micro	Macro	Micro
Non-colitic	0*	0*	0*	0*	0^∗#^	0^∗#^
TNBS control	6 (1.5)	18 (2)	6 (1.5)	19 (2.5)	5.5 (1.75)^#^	18.5 (1)^#^
EC 10	4 (1)*	14 (1.5)*	2 (1)*	13 (2)*	3 (1)*	15 (2)*
EC 50	4 (2.5)	16 (2.5)	3 (1)	15 (1.5)	4 (1.5)^#^	16 (1.5)
Sulfasalazine	3.5 (2.5)	15.5 (2.5)	4 (1.5)	16 (1.5)	5 (2)^#^	17 (2)^#^
Not relapsed TNBS	—	—	—	—	2 (1)*	12 (1)*

Results expressed as median (interquartile range). Kruskal-Wallis-Mann-Whitney, *P < 0.05 comparing to TNBS group, ^#^ *P* < 0.05 comparing to not relapsed TNBS control. *n* = 4–7. EC 10 and 50: Epicatechin, dose 10 and 50 mg/kg.

**Table 5:** Biochemical quantifications of glutathione, myeloperoxidase, and alkaline phosphatase in the colon of animals submitted to the chronic colitis model.

	Glutathione (GSH) (nmol/g)		
	GSH 1st week	GSH 2nd week	GSH 3rd week
Non-colitic	1,727.88 ± 95.34*	1,134.20 ± 79.27	1,726.73 ± 34.92^∗#^
TNBS control	1,323.49 ± 43.15	1,353.74 ± 77.53	1,374.42 ± 77.55^#^
EC 10	1,662.56 ± 99.92*	1,236.37 ± 45.62	1,673.06 ± 82.37^∗#^
EC 50	1,529.50 ± 87.26	1,150.68 ± 52.38	1,658.92 ± 110.49
Sulfasalazine	1,555.76 ± 141.52	1,269.79 ± 57.53	1,562.79 ± 110.64^#^
Not relapsed TNBS	—	—	1,914.64 ± 63.62*

	Myeloperoxidase (MPO) (U/g)		
	MPO 1st week	MPO 2nd week	MPO 3rd week

Non-colitic	64.56 ± 19.05*	101.65 ± 6.63*	71.69 ± 5.64^∗#^
TNBS control	377.52 ± 85.14	219.49 ± 25.09	634.42 ± 149.44^#^
EC 10	386.31 ± 76.80	247.64 ± 34.71	401.91 ± 70.28
EC 50	463.36 ± 98.97	211.67 ± 51.93	504.98 ± 144.73
Sulfasalazine	383.79 ± 81.39	179.12 ± 26.34	450.46 ± 73.96^#^
Not relapsed TNBS	—	—	225.23 ± 46.09*

	Alkaline phosphatase (ALP) (mU/mg of protein)		
	ALP 1st week	ALP 2nd week	ALP 3rd week

Non-colitic	20.93 ± 2.21*	28.18 ± 1.56*	17.56 ± 1.39*
TNBS control	46.94 ± 2.62	40.02 ± 3.63	27.39 ± 3.76
EC 10	54.04 ± 6.67	34.96 ± 1.56	27.23 ± 2.10
EC 50	49.89 ± 7.95	48.16 ± 9.97	30.31 ± 1.20
Sulfasalazine	55.92 ± 7.85	39.40 ± 5.07	38.38 ± 4.96^#^
Not relapsed TNBS	—	—	24.25 ± 2.71

Results expressed as mean ± E.P.M. ANOVA-Dunnet comparing to the checks of each group. *P < 0.05 comparing to TNBS control, ^#^ *P* < 0.05 comparing to not relapsed TNBS control. *n* = 4–7. EC 10 and 50: Epicatechin, dose 10 and 50 mg/kg.

**Table 6:** Immunohistochemical quantifications of cyclooxygenase-2 (COX-2) and poliferating cell nuclear antigen (PCNA) and immunoblotting of epidermal growth factor (EGF) and inducible nitric oxide sintase (iNOS) in the colon of the animals submitted to the chronic colitis model.

	COX-2 (*μ*m^2^/mm^2^)	PCNA (cells/mm^2^)	EGF (RU)	iNOS (RU)
1st week				
Non-colitic	13,151.38 ± 7,642.79*	277.94 ± 21.54*	73.50	62.68
TNBS control	42,561.22 ± 7,504.32	452.82 ± 74.51	156.94	199.82
EC 10	8,515.84 ± 7,309.41*	568.37 ± 20.43*	298.14*	166.03
Sulfasalazine	16,594.99 ± 6,111.12*	387.24 ± 23.76	139.87	132.46
2nd week				
Non-colitic	5,899.88 ± 6,947.30*	274.82 ± 28.95*	66.47	53.30
TNBS control	52,894.12 ± 10,978.02	371.63 ± 25.49	98.12	258.84
EC 10	8,599.16 ± 7,965.00*	306.04 ± 19.91	225.38*	195.38
Sulfasalazine	29,415.99 ± 11,128.13	396.61 ± 22.43	82.07	271.02
3rd week, relapse				
Non-colitic	6,618.02 ± 7,509.17*	237.34 ± 30.82*	105.70	92.57
TNBS control	46,529.49 ± 11,713.00	359.13 ± 16.83	131.66	180.48
EC 10	8,139.53 ± 8,095.28*	484.05 ± 21.86^∗#^	201.54*	198.47
Sulfasalazine	16,125.29 ± 10,938.09	412.22 ± 28.92^#^	94.62	147.59
Not relapsed TNBS	16,986.12 ± 8,481.50	306.04 ±23.26	—	—

Results expressed as mean ± E.P.M. ANOVA-Dunnet, *P < 0.05 comparing to the TNBS group, ^#^ *P* < 0.05 comparing to not relapsed TNBS control. *n* = 4–7. EC 10: Epicatechin, dose 10 mg/kg. RU: random units.
